# Supplementary figures and images for: Analysis of the Drosophila Ajuba LIM protein defines functions for distinct LIM domains
Source: PLoS One. 2022 Aug 15;17(8):e0269208. doi: 10.1371/journal.pone.0269208 (PMC9377591; doi:10.1371/journal.pone.0269208)

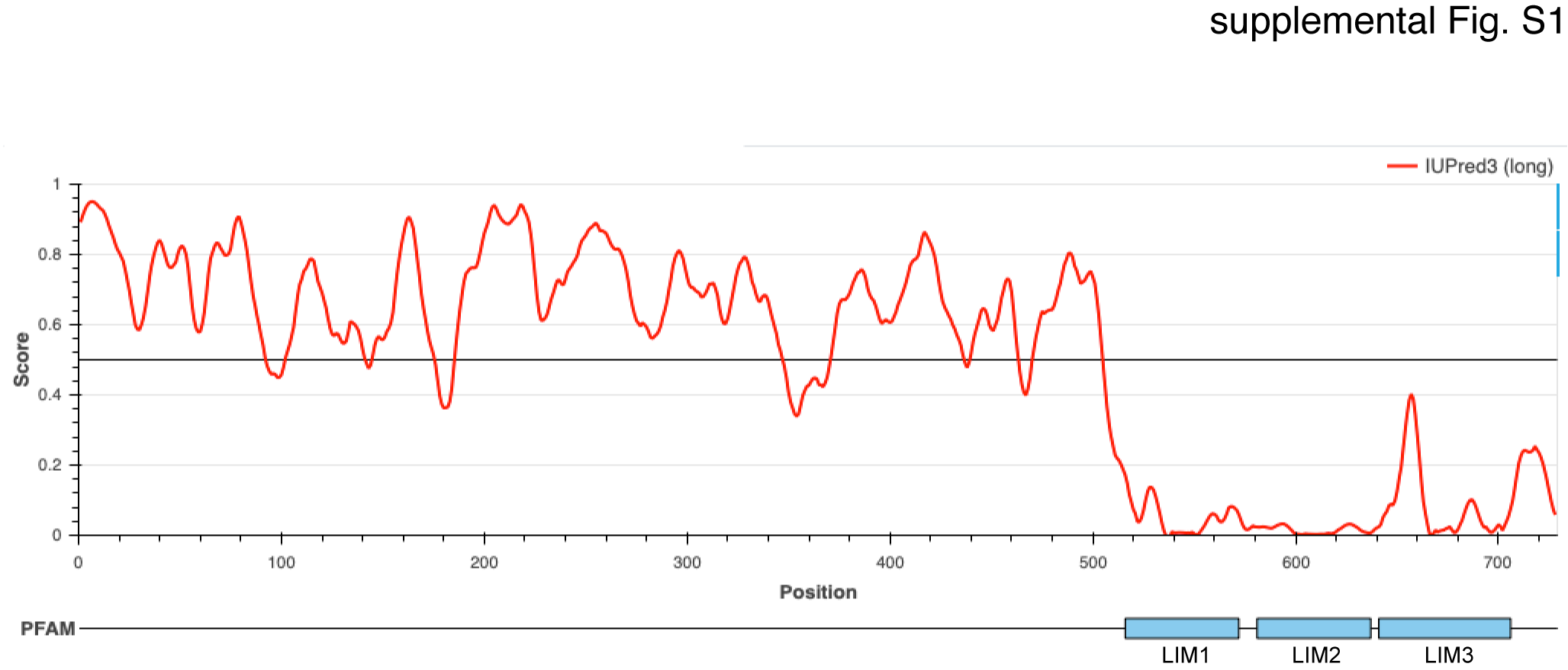

Supplement: S1 Fig — The probability that each residue in Jub is part of an intrinsically disordered region, as calculated using IUPred3 [44], is displayed. The positions of the three LIM domains are also indicated. (TIF) [file pone.0269208.s001.tif]

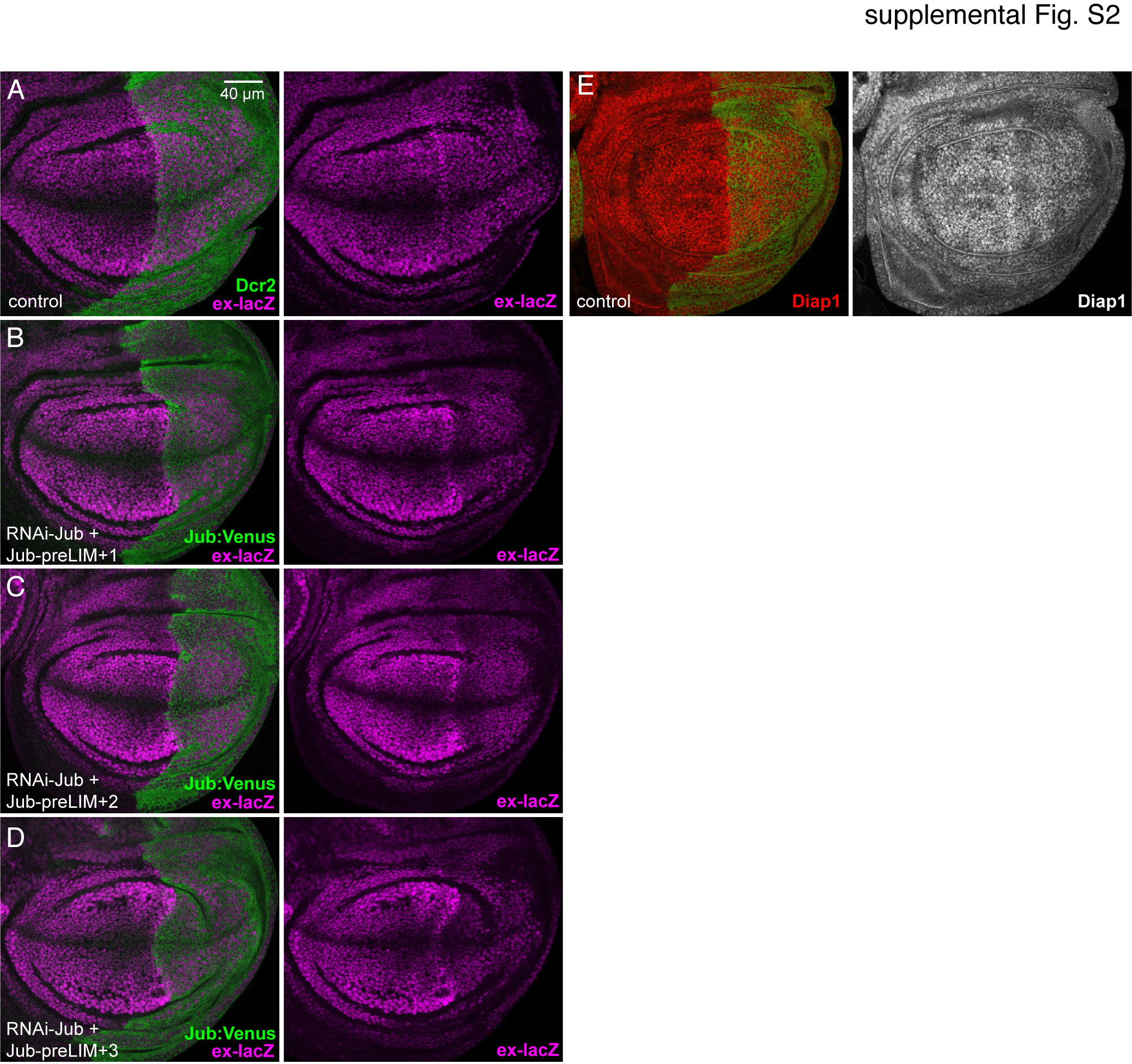

Supplement: S2 Fig — A-D) Representative wing discs from larvae expressing ex-lacZ (magenta), hh-Gal4 UAS-Dcr2 and A) control B) UAS-RNAi-jub UAS-Jub-preLIM+1:msVenus. C) UAS-RNAi jub UAS-Jub-preLIM+2:msVenus. D) UAS-RNAi jub UAS-Jub-preLIM+3:msVenus. All discs are shown at the same magnification (scale bar = 40 μm). E) Representative wing disc stained for Diap1 (red/white) from larvae expressing hh-Gal4 UAS-Dcr2, shown at the same magnification as for ex-lacZ stains. (TIF) [file pone.0269208.s002.tif]
