# Supplementary material for: Analysis of the Drosophila Ajuba LIM protein defines functions for distinct LIM domains
Source: PLoS One. 2022 Aug 15;17(8):e0269208. doi: 10.1371/journal.pone.0269208 (PMC9377591; doi:10.1371/journal.pone.0269208)

S3 raw images for Fig. 1J

Fig 1J top blot. input,mouse anti-V5, anti-mouse IgG-800

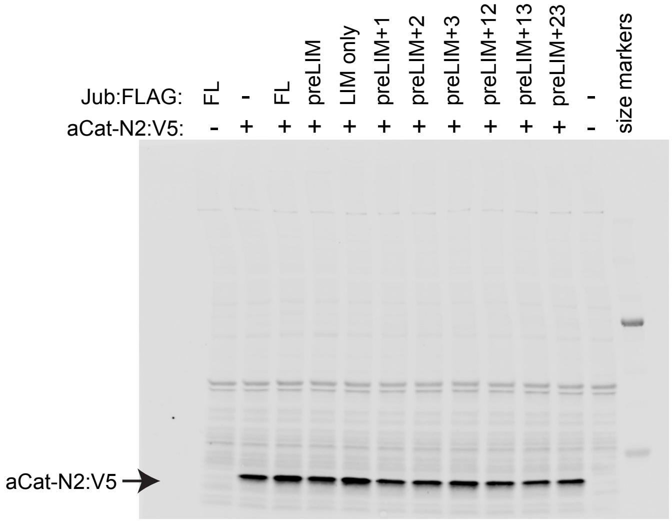

Fig 1J 4th from top blot. IP V5,mouse anti-V5, anti-mouse IgG-800

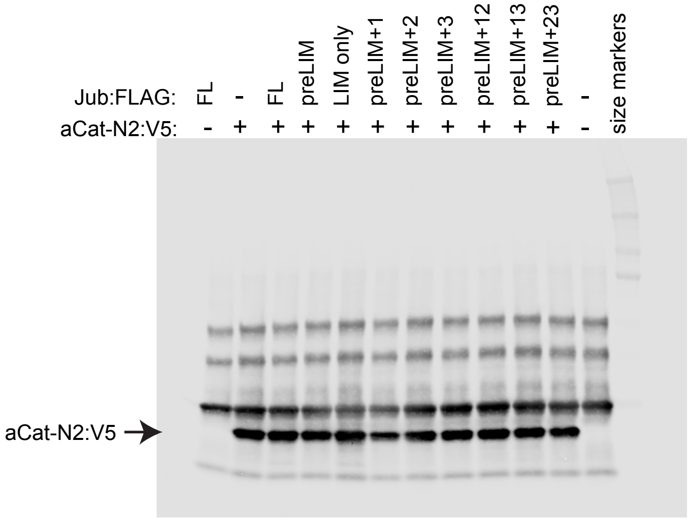

Fig 1J 2nd from top blot. input, rabbit anti-FLAG, anti-rabbit IgG 680

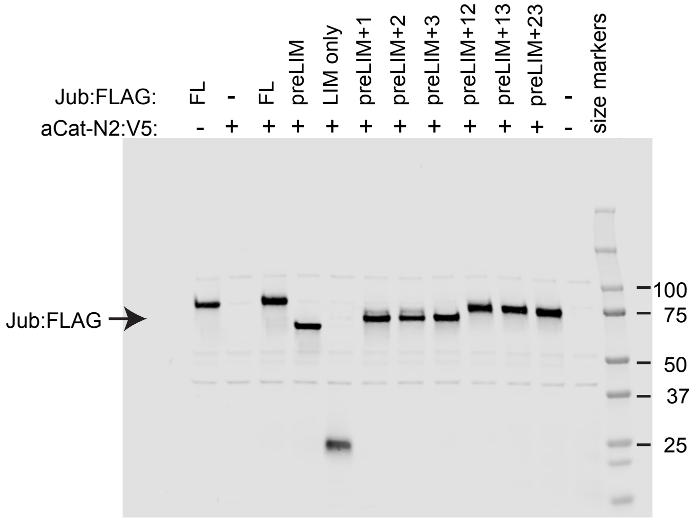

Fig 1J bottom blot. IP V5, rabbit anti-FLAG, anti-rabbit IgG 680

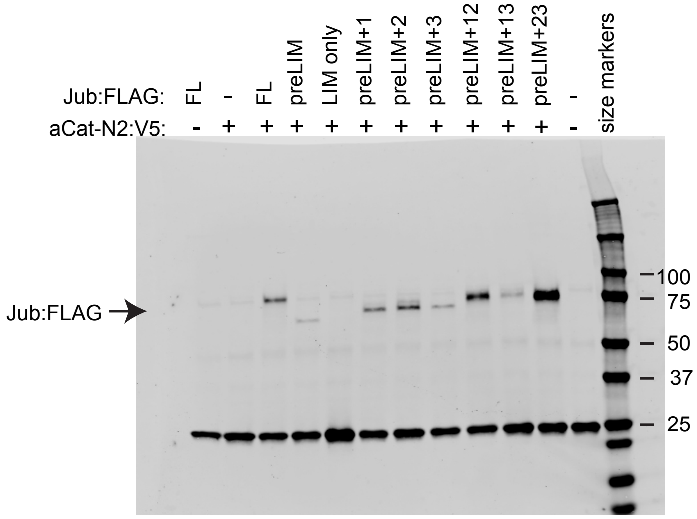

Fig 1J 3rd from top blot. input, mouse anti-GAPDH, anti-mouse IgG 680

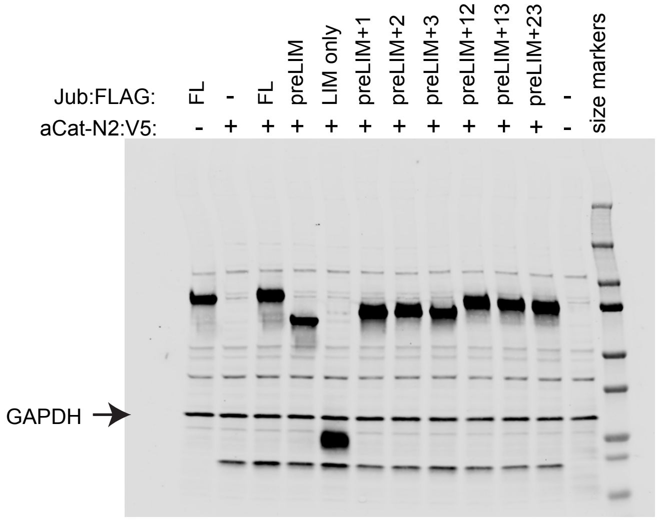

(blot above was reblotted, bands from initial blotting are still visible)

S3 raw images for Fig. 5A

Fig 5A top blot. input,mouse anti-V5, anti-mouse IgG-800

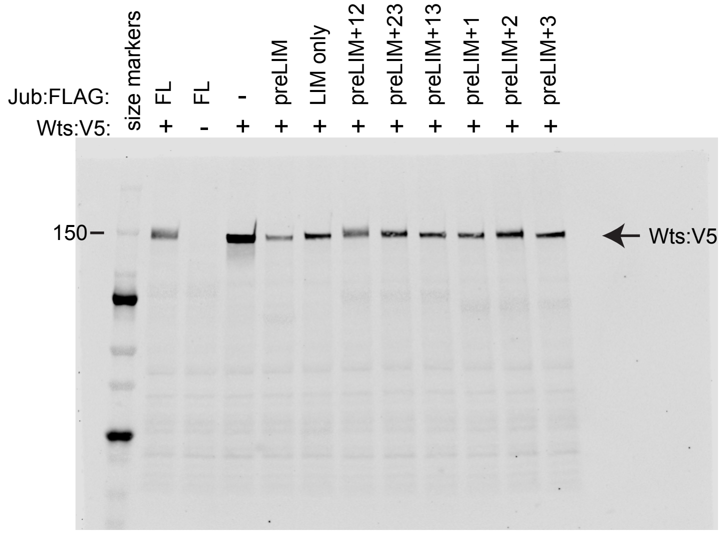

Fig 5A 4th from top blot. IP V5,mouse anti-V5, anti-mouse IgG-800

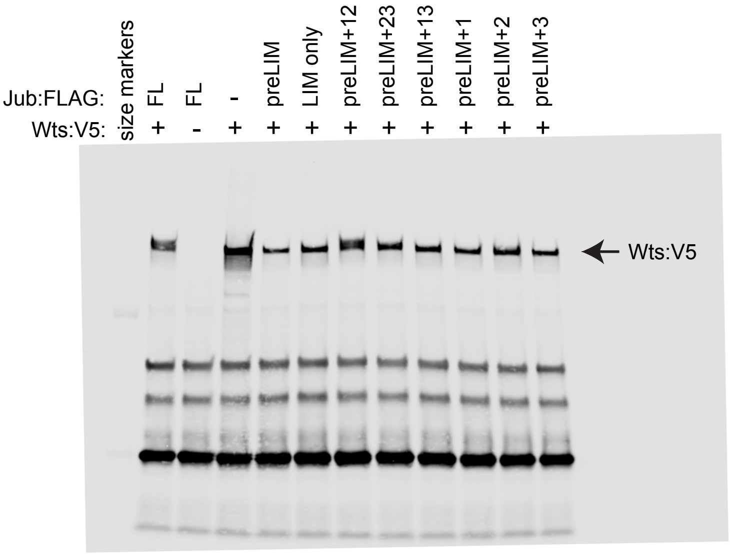

Fig 5A 2nd from top blot. input, rabbit anti-FLAG, anti-rabbit IgG 680

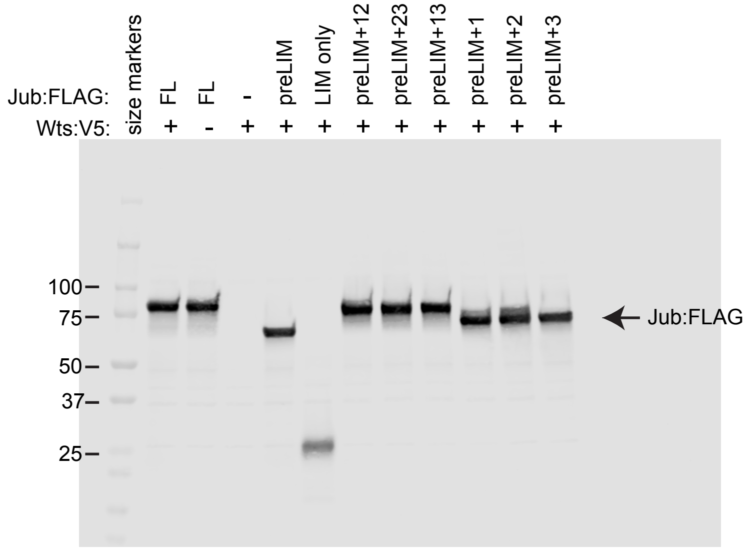

Fig 5A bottom blot. IP V5, rabbit anti-FLAG, anti-rabbit IgG 680

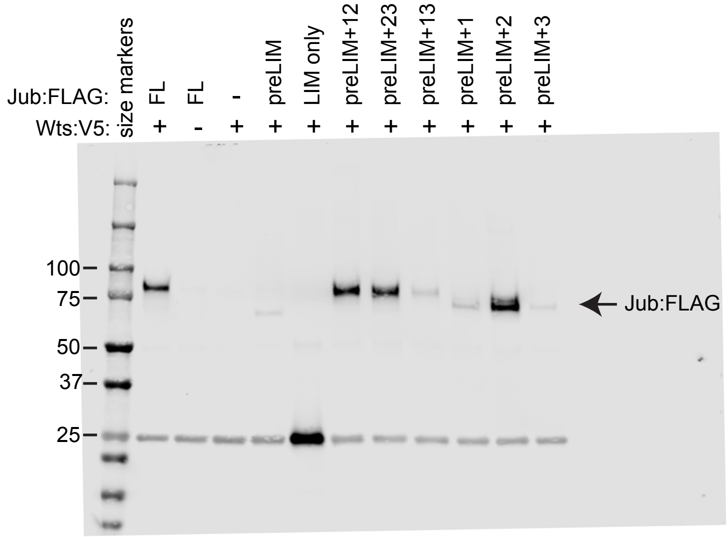

Fig 5A 3rd from top blot. input, mouse anti-GAPDH, anti-mouse IgG 680

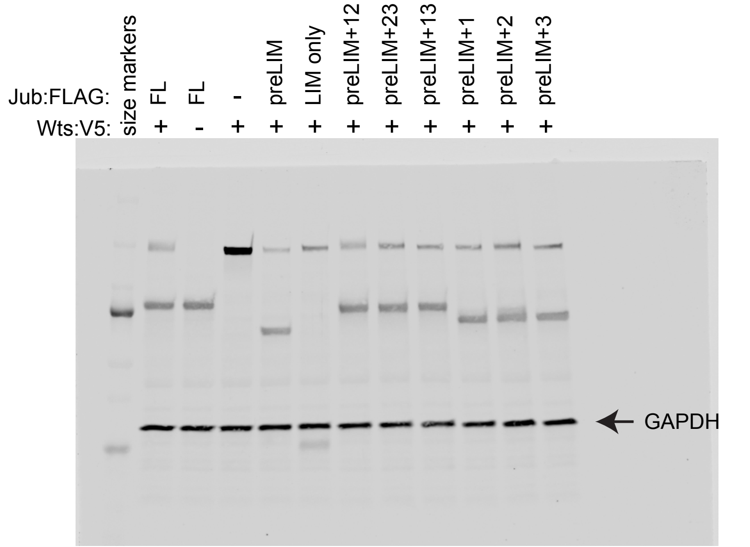

(blot above was reblotted, bands from initial blotting are still visible)

S3 raw images for Fig. 5B

Fig 5B top & 3rd blots. input,mouse anti-V5, mouse anti-GAPDH anti-mouse IgG-800

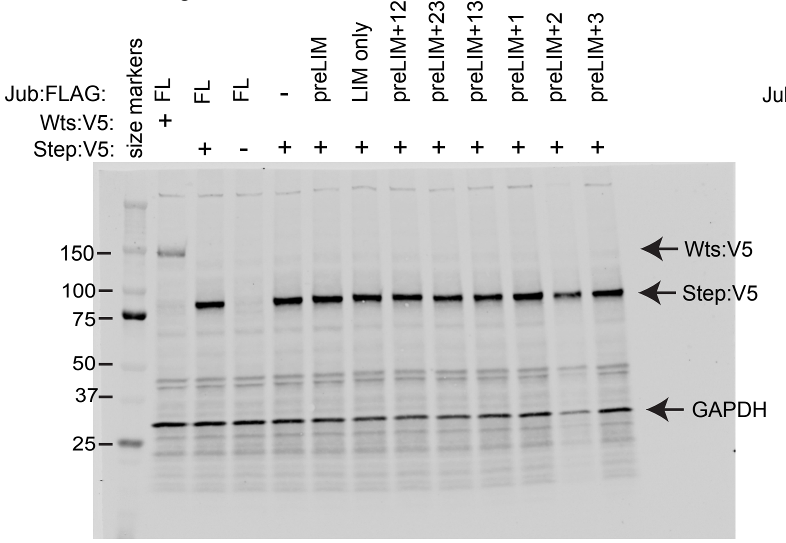

Fig 5B 4th from top blot. IP V5,mouse anti-V5, anti-mouse IgG-800

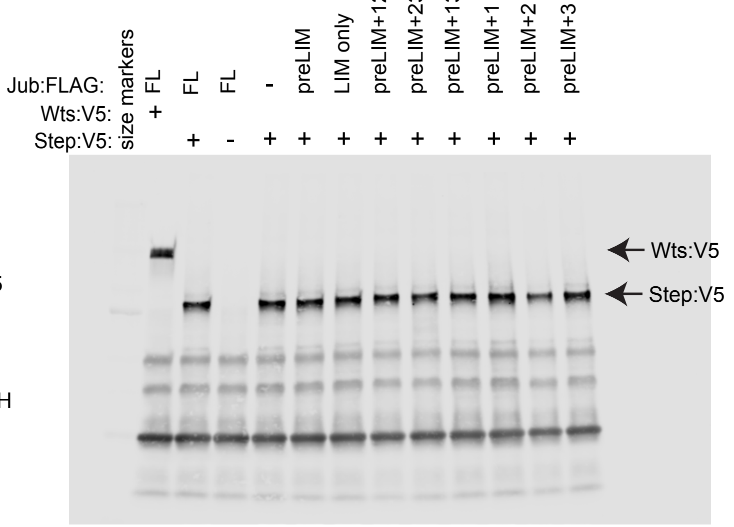

Fig 5B 2nd from top blot. input, rabbit anti-FLAG, anti-rabbit IgG 680

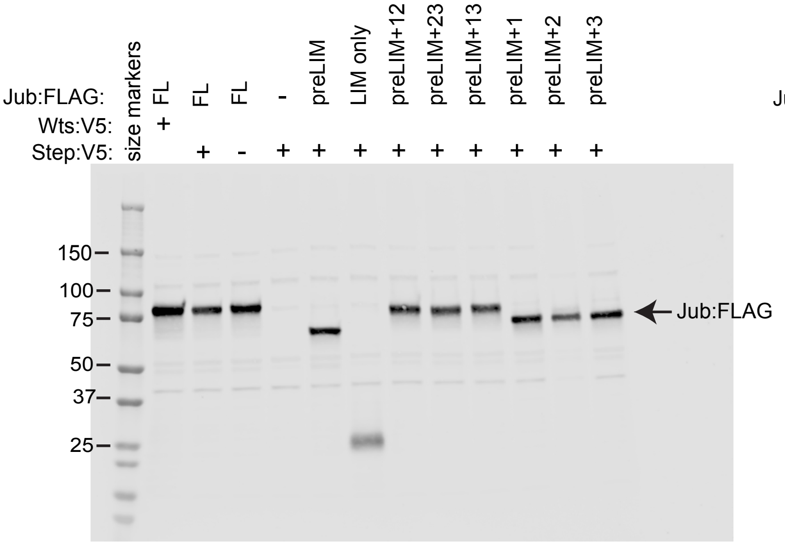

Fig 5B bottom blot. IP V5, rabbit anti-FLAG, anti-rabbit IgG 680

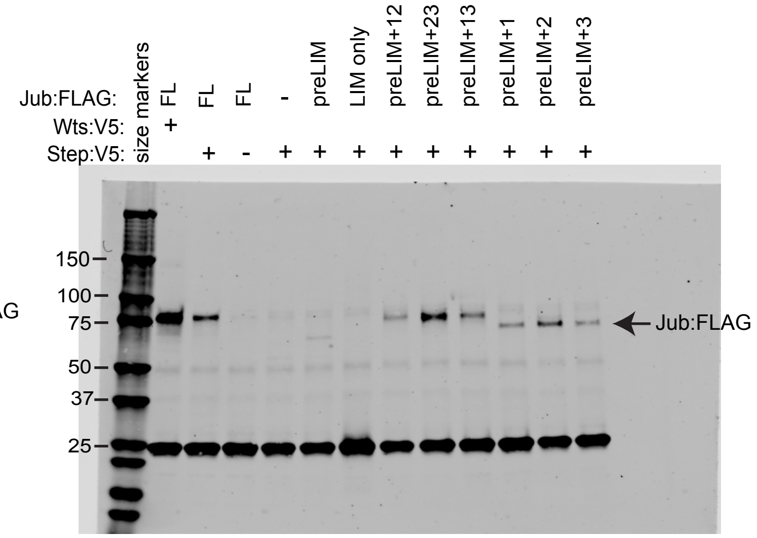

Supplement: S1 Raw images — (PDF) [file pone.0269208.s003.pdf]
